# Ulcerative Endocarditis

**Published:** 1891-04-04

**Authors:** 


### ULCERATIVE ENDOCARDITIS.

This affection has attracted the attention of pathologists and clinicians, and the views held in relation to it, once so conflicting and divergent, have been surely, though gradually, merging, till now it is generally regarded as infective in origin, and in its infective origin differing essentially from the ordinary varieties of endocarditis, the result of inflammation from various causes other than microbal. Few, indeed, will be prepared to adopt the views of M.

Germain Sée, who regards all forms of endocarditis alike, the acute, subacute, vegetative and ulcerative, as invariably parasitic, and therefore denies the purely inflammatory origin of any form. According to M. Sée, the endocardium is often affected by the latent microbe of a past and sometimes long-forgotten attack of enteric or scarlet fever, diphtheria, pneumonia, or syphilis. One form he does except from this bacteriological origin—viz., the chronic valvulitis, so frequently found in the aortic valves of the aged, and associated with atheromatous arteries. It is certain, however, that malignant or ulcerative endocarditis is parasitic in origin. Dr. Frederick Taylor, in his paper on "Ulcerative Endocarditis," read before the Metropolitan Counties Branch of the British Medical Association on February 4th, reminds us of the observations of M. Lion and M. Martineau, according to whom the organisms of septicæmia and pyæmia, pneumonia, typhoid fever, and of tuberculosis, as well as six other varieties of micro-organisms not hitherto associated with any particular diseases, had all been found in cases of septic endocarditis. Dr. Pitt also exhibited some microscopical specimens showing the presence of organisms in ulcerative endocarditis at this meeting. Dr. John Ely has reported a case of much interest, of a patient who had died of malignant endocarditis. In his case none of the ordinary sources of infection could be discovered, but the urethra being examined, pus was found in the passage. A microscopic examination of this part and of the pus resulted in the discovery of chains and clusters of micrococci, which were similar in appearance and in their reaction to staining reagents to those found in the internal organs. In this case it was thought that the ulcerative endocarditis, and the accompanying pathological changes in the other organs, were attributable to the presence of the staphylococcus pyogenes aureus, and that the probability was that these micro-organisms gained access through the mucous membrane of the urethra. A point of great clinical interest, to which Dr. Taylor draws attention, is that in the various diseases with which any case of ulcerative endocarditis is associated, the course and complications may be differentiated. He instanced the fact noted by several observers, that septic endocarditis following pneumonia had an especial tendency to attack the aortic valves, and sometimes was associated with suppurative meningitis.

